# Psychometric assessment of the Persian version of the DUSAETCS questionnaire: Duke University Self-Assessment Educational Technology Competencies

**DOI:** 10.1371/journal.pone.0360635

**Published:** 2026-11-06

**Authors:** Haniyeh Rezaie, Aeen Mohammadi, Rita Mojtahedzadeh, Maryam Aaalaa, Nikoo Yamani

**Affiliations:** 1 Department of Medical Education, School of Medicine, Tehran University of Medical Sciences, Tehran, Iran; 2 Intelligent and Virtual Technologies in Learning Research Lab, School of Medicine, Tehran University of Medical Sciences, Tehran, Iran; 3 Medical Education Research Center, Medical Education Development Center (MEDC), Isfahan University of Medical Sciences, Isfahan, Iran; Smart University of Medical Sciences, IRAN, ISLAMIC REPUBLIC OF

## Abstract

**Introduction:**

The rapid global evolution of Information and Communication Technologies (ICT), combined with the shift toward e-learning in higher education, has made it more necessary than ever to equip faculty members with technological skills. The level of self-efficacy significantly influences technological competence in educational technology. This study aimed to translate and conduct a psychometric evaluation of the Persian version of the Duke University School of Nursing's self-assessment of educational technology competencies: DUSAETCS for faculty members.

**Method:**

The psychometric properties of the DUSAETCS questionnaire, comprising 44 items, were evaluated. The original questionnaire was translated into Persian via back translation. Internal and external consistencies, factor analysis, and construct validity analysis were examined.

**Results:**

Of the initial 44 items, 26 were retained following the assessment of content validity using the Content Validity Ratio (CVR) and Content Validity Index (CVI). The overall CVI and CVR were 0.79 and 0.99, respectively. The Persian version of the questionnaire demonstrated high internal consistency, with a Cronbach’s alpha coefficient of 0.92 for the total scale.

**Conclusions:**

The findings provide preliminary evidence supporting the content validity and internal consistency of the Persian version of the DUSAETCS for assessing faculty members’ educational technology competencies in the context of e-teaching. Further psychometric evaluation, particularly assessment of construct validity and other reliability measures, is recommended before broader application of the instrument.

The pervasive desire to learn in online environments has gained renewed significance in recent years, particularly with the increasing integration of technology into education [1]. E-learning has garnered increasing attention, as technological advancements have become an integral part of learners’ lives and provided medical educators with a versatile toolkit [2]. Higher education institutions are recommended to adopt a virtual delivery mode for in-person and traditional courses [3]. Furthermore, a need to reconceptualize teaching methods and create online and hybrid learning environments [4,5] has prompted researchers to focus on teachers’ digital literacy. However, faculty members’ readiness and willingness to adopt virtual teaching have been reported to vary across studies [3,6,7] and the factors influencing this variability remain underexplored Simply offering a traditional training course online is not an effective strategy. Virtual training becomes effective when the following points are taken into consideration: The teacher should actively provide the following measures and conditions: Design and present a high-quality course [8,9], implement appropriate assessment strategies [10], and foster collaboration and interaction among learners [11].

Adhering to the mentioned points is challenging for some faculty members. These challenges are related to the separation between the instructor and the learners [12] or the lack of virtual teaching experience [13]. The separation between teachers and learners in virtual teaching has led to a problem for teachers to struggle to communicate effectively with learners, and they are unable to extend their abilities from face-to-face classes to virtual ones [14]. For instance, teachers in face-to-face classes can enhance teacher-learner communication by using facial expressions and body language, which in an online environment relies more on voice communication [15]. In addition, compared to face-to-face teaching, teachers reported spending more time adopting to the online teaching environment, designing ways to interact with learners, and improving learners’ understanding of the educational content [16]. Therefore, it is essential to acknowledge that several factors contribute to faculty career success, including self-efficacy. Self-efficacy influences how individuals perceive themselves, think, and motivate themselves to engage in specific behaviors. Studies also show that faculty members’ competence in educational technology is influenced by levels of self-efficacy [17].

Self-efficacy is a strong predictor of the activity individuals intend to pursue, leading to increased persistence and motivation in performance [18]. From a social cognitive point of view, the self-efficacy structure shows a person's belief in their ability to perform daily tasks, which leads to their decision-making in matters [18]. Thus, self-efficacy is a crucial concept for understanding how individuals feel and perform in their work [19]. According to Bandura (1997), individuals with high self-efficacy view challenges as opportunities, exhibit strong commitment to the recovery tasks, invest significant time and effort, demonstrate strategic problem-solving skills, bounce back quickly from failures, and feel in control [18]. Teachers with high self-efficacy tend to engage in more thorough planning and organization [20]. They are also more receptive to new ideas and willing to experiment with different methods to better address their students’ needs [21,22]. As a result, their willingness to adopt new teaching methods and technologies increases.

Accordingly, teachers’ self-efficacy plays a vital role in the extent to which they exhibit sustainability when facing unforeseen issues and in the strength of their motivation to engage in specific teaching behaviors, such as the use of digital educational tools [23]. Teachers with higher self-efficacy are more involved with learners and experience more job satisfaction [24]. They also tend to be more persistent in the face of educational problems and try more creative strategies to help learners understand complex topics [25]. Additionally, teachers’ higher self-efficacy in educational technology positively affects their teaching ability [26]. Hence, having an appropriate instrument to assess faculty members’ perceived competencies in the effective use of educational technology is important for identifying areas requiring further professional development.

Several instruments have been developed to assess competencies or self-efficacy related to online teaching. Among them, the Online Teaching Self-Efficacy Inventory (OTSEI) assesses different aspects of online teaching, including the selection of technological resources, virtual interaction, migration of course content, online course alignment, and web-based course structure [7]. However, because the present study focused specifically on faculty members’ perceived competencies in the pedagogically appropriate use of educational technologies, the Duke University School of Nursing Self-Assessment of Educational Technology Competencies Scale (DUSAETCS) was selected. The DUSAETCS was developed by Valiga and the Duke University School of Nursing Institute for Educational Excellence to assess faculty members’ educational technology competencies and identify their professional development needs. The instrument addresses competencies related to selecting and evaluating educational technologies, developing technology-supported learning experiences, promoting student learning through technology, applying principles of effective teaching, and using digital educational tools [27].

The DUSAETCS has previously been used among nursing educators to examine self-assessed educational technology competence and its relationship with online teaching self-efficacy; however, further research has been recommended to establish its psychometric properties [28].

Although e-learning has become increasingly common in Iranian higher education, concerns remain regarding faculty members’ readiness and confidence in using educational technologies effectively. Previous studies in Iran have mainly focused on general e-learning readiness, attitudes toward online education, and technological, pedagogical, and content knowledge. For example, Farazkish and Montazer (2019) [29]. reported relatively low levels of e-learning readiness among faculty members from 23 Iranian universities, while Naghashpour et al (2023) also highlighted the need to strengthen technological and pedagogical competencies among medical faculty [30]. More recently, virtual teaching self-efficacy has been examined among Iranian medical educators; however, the available studies have generally relied on broader measures of teaching self-efficacy rather than instruments specifically designed to assess educational technology competencies in virtual teaching [31].Therefore, there is still limited evidence on a culturally adapted and psychometrically validated Persian instrument that can comprehensively assess these competencies among faculty members. Addressing this gap may help identify specific professional development needs and support more effective use of educational technologies in Iranian medical universities.

## Methods

### Study design and participants

This study is methodological (psychometric) research aimed at assessing the validity and reliability of the questionnaire, conducted at Tehran University of Medical Sciences in Iran. The consent of the original questionnaire’s author had previously been obtained from Duke University by Richter [28]. This psychometric assessment study was carried out in two phases: 1) the translation-back translation, and 2) assessment of the validity and reliability of the questionnaire. The translation was executed following the recommended International Test Commission (ITC) for test translation and adaptation (2nd edition) [32]. Data were collected between July and September 2024 using convenience sampling. The target population included faculty members affiliated with Iranian universities of medical sciences. Inclusion criteria included: being a faculty member of an Iranian university of medical sciences, having at least one semester of online or virtual teaching experience, and providing informed consent to participate. Participants who withdrew their consent before completing the questionnaire or submitted substantially incomplete questionnaires were excluded from the analysis. Potential participants were invited via email and professional groups on WhatsApp and Telegram. The invitation included a brief explanation of the study and a link to the web-based Persian DUSAETCS system. A total of 92 eligible faculty members submitted complete questionnaires and were included in the final analysis.

### Research instrument

DUSAETCS was provided by faculty members in the Duke School of Nursing’s Institute for Educational Excellence as a self-assessment tool for new faculty to assess their technology competence and facilitate faculty members’ development [27]. The tool was based on Chickering and Gamson’s seven principles for good practice in undergraduate education [33]. It contains 44 items with Likert-scale responses ranging from 1 (not at all competent) to 4 (very skilled). Four subscales are shown in Table 1. The original version has been validated as a highly reliable and valid tool for assessing faculty members’ technology competence and supporting faculty members’ development, as demonstrated by Richter [28].

**Table 1:** Subscale of DUSAETCS questionnaire.

	Area	Number of Items
1	Competency	**11**
2	Effectively Use Educational Technologies to Help Students Achieve Outcomes	**14**
3	Use Educational Technologies to Implement Principles of Good Teaching Effectively	**7**
4	Create Effective Learning Experiences	**12**

### Translation and psychometric evaluation of the instrument

The psychometric evaluation of the Persian version of the DLRQ comprised the following steps

#### Step 1: Translation process.

In this phase, the questionnaire was translated into Persian using the forward-backward translation technique recommended by the World Health Organization [34]. Initially, each item of the English questionnaire was translated into Persian by two academic experts in English translation. Second, two bilingual university instructors examined each translated item for meaning, accuracy, wording, spelling, and grammar. Subsequently, a back-translation into English and a comparison of the original questionnaire with the back-translated version were conducted. The results indicate the accuracy of the primary translators’ performance. The final Persian version of the questionnaire consisted of 44 questions, each on a 4-point Likert scale (1 (not at all competent) to 4 (very skilled)).

#### Step 2: Content validity.

The content validity of the initial DUSAETCS was investigated both quantitatively and qualitatively by expert opinion. Six faculty members were recruited based on their experience in online and technology-enhanced learning and their expertise in medical education. They were asked to evaluate each item in the DUSAETCS against the criteria of “essential,” “relevance,” “clarity,” and “simplicity.” Each item was assessed using Likert scales: A three-point scale for “essential” (1– unessential, 2 – helpful, but not essential, and 3 – essential,), and four-point scales for “relevance” (1 – not relevant, 2 – rather appropriate, 3 – relevant, and 4 – completely relevant) and “clarity” (1 – not simple, 2 – relatively simple, 3 – simple and 4 –straightforward) criteria. In addition, the experts were asked to provide comments on the “simplicity” of each item (fluency and the use of simple, understandable words) and on the most appropriate placement and order of the items. The Content Validity Ratio (CVR) was calculated from the experts’ ratings of essentiality, whereas the item-level Content Validity Index (I-CVI) was calculated from ratings of relevance. Comments regarding clarity, simplicity, wording, cultural applicability, and item order were used for qualitative revision. They suggested adding an item titled ‘I can create effective learning experiences using podcasts’ in the field of creating practical learning experiences.

For a panel of six experts, the critical CVR value was 0.99. Therefore, an item met the CVR criterion only when all six experts rated it as essential, producing an observed CVR of 1.00. An I-CVI criterion of 0.78 was applied; with six experts, this required at least five experts to rate an item as relevant, corresponding to an observed I-CVI of at least 0.83. CVR and I-CVI were interpreted exclusively as indices of content validity and not as measures of reliability.

#### Step 3: Construct validity.

Construct validity was initially examined using exploratory factor analysis (EFA). Principal axis factor analysis was applied with Varimax rotation [35]. Sampling adequacy and factorability of the correlation matrix were assessed using the Kaiser-Meier-Olkin (KMO) criterion and Bartlett’s test of sphericity.

Following the exploratory factor analysis, confirmatory factor analysis (CFA) was conducted using IBM SPSS AMOS version 24 to provide an initial assessment of the five-factor measurement model. The model included five first-order factors: instructional use of educational technology, good teaching practices, proficiency in educational technology tools, educational technology competence, and technology-enhanced learning resources. Since the dimensions of instructional use of educational technology and good teaching practices were conceptually related, a higher-order factor was identified to represent their common underlying structure while maintaining them as distinct first-order factors. Model fit was assessed using the chi-square statistic, the ratio of chi-square to degrees of freedom (χ^2^/df), comparative fit index (CFI), Tucker-Lewis index (TLI), root mean square error of approximation (RMSEA) with 90% confidence interval, root mean square residual (RMR), goodness of fit index (GFI), and adjusted goodness of fit index (AGFI). Standardized factor loadings were examined for all observed items. Composite reliability (CR) and average variance extracted (AVE) were used to assess construct reliability and convergent validity, respectively. Since the same sample was used for EFA and CFA, CFA was considered as a preliminary confirmation rather than an independent factor structure.

#### Step 4: Reliability assessment.

Internal consistency reliability was assessed using Cronbach’s alpha. Alpha coefficients were calculated for the total Persian DUSAETCS and separately for the four original content domains using responses from the 92 participating faculty members. A coefficient of 0.70 or higher was considered acceptable. In addition, the reliability of the five latent factors identified in the CFA was evaluated using composite reliability. Because the questionnaire was administered only once, test–retest reliability and the intraclass correlation coefficient (ICC) were not assessed.

### Ethical consideration

Ethics approval for this study was obtained from the Research and Technology Department of Tehran University of Medical Sciences (ID IR.TUMS.VCR.REC.1400.1290). The purpose and procedures of the study were explained to all participants, including medical education experts and faculty members involved in virtual education. Participation was voluntary, and participants were free to decline participation or withdraw from the study at any time without any consequences. Informed consent was obtained electronically from all participants. An informed consent statement was presented at the beginning of the online questionnaire, and participants provided their consent by proceeding to complete the questionnaire.

## Results

### Demographic data

Medical Sciences faculty members consisted of 71 females and 21 males, with 54% assistant professors and 23% associate professors.

### Content and face validity

The experts confirmed the instrument’s face validity. Regarding content validity, the questions with a CVR of 99% and a CVI of at least 79% were confirmed in the questionnaire. Out of the initial 44 items, 26 items remained after calculating the Content Validity Ratio (CVR) and Content Validity Index (CVI). The excluded items either overlapped with other items or were deemed culturally irrelevant or inapplicable in our country. Table 2 presents the CVI and CVR for each questionnaire item.

**Table 2:** CVR and CVI of DUSAETCS.

Questions	Area of competency	Content Validity Index	Content Validity Ratio
Item 1	Locate, analyze, and evaluate innovative educational technologies for their appropriate use in the courses I teach and projects in which I am involved	67	67
Item 2	Develop learning experiences that take full advantage of what innovative educational technologies have to offer	100	100
Item 3	Develop technology-enriched learning environments that enable students to pursue their individual curiosities, set their own educational goals, manage their own learning, and assess their own progress	50	0
Item 4	Develop and test creative applications of technology tools and techniques in the evaluation of student learning	100	100
Item 5	Collaborate with students, peers, and experts – in the subject, education, and technology – to design and implement digital tools and resources that enhance learning and professional development	67	33
Item 6	Use research-based evidence to guide decisions concerning whether and how I incorporate educational technology in the courses I teach and projects in which I am involved	100	100
Item 7	Use research-based evidence to guide decisions concerning whether and how I incorporate educational technology in the courses I teach and projects in which I am involved	100	100
Item 8	Help students and faculty colleagues use digital tools and resources to enhance their ongoing personal and professional development	67	0
Item 9	Exhibit leadership by actively participating in community building and advancing a spirit of innovation related to the use of educational technology	50	33
Item 10	Participate in groups and communities to explore innovative use of technology to improve student outcomes	50	33
Item 11	Considered the financial implications of using technology-based tools and techniques in my courses	67	33
**I can effectively use educational technologies to help students achieve the following outcomes:**			
Item 12	Think deeply	100	100
Item 13	Solve real-world problems	67	0
Item 14	Engage in the discussion of “thorny” issues	100	100
Item 15	Exhibit cultural humility/sensitivity	67	66
Item 16	Reflect on personal values and how they influence one’s actions	83	100
Item 17	Communicate effectively … with peers, patients, families, nurse colleagues, and other members of the healthcare team	67	66
Item 18	Function as a leader/evolving leader	67	0
Item 19	Function as a scholar/evolving scholar	83	100
Item 20	Manage information effectively	83	100
Item 21	Be creative and innovative	100	100
Item 22	Know one’s own strengths and areas needing improvement	100	100
Item 23	Incorporate a community/population/global perspective	67	0
Item 24	Appreciate the strength in diversity	67	0
**I can use educational technologies to effectively implement principles of good teaching (Chickering & Gamson, 1987)**			
Item 25	Provide meaningful contact between students and faculty	83	100
Item 26	Encourage reciprocity and cooperation among students	100	100
Item 27	Engage students in active learning	83	100
Item 28	Provide prompt feedback to students	100	100
Item 29	Provide for appropriate time on course tasks/projects/assignments	100	100
Item 30	Communicate high expectations to students	83	100
Item 31	Respect the diverse talents and ways of learning represented in the student group	67	66
**I can create effective learning experiences using the followings:**			
Item 32	Narrated Slides (MS PowerPoint)	100	100
Item 33	Discussion forums (LMS Discussion Board tools)	100	100
Item 34	Collaborative student project tools On-site studio-based video Narrated PowerPoint Box/MS OneDrive Adobe Premiere	67	33
Item 35	Virtual storage and file management for large project files	100	100
Item 36	Web Conferencing tools	100	100
Item 37	Video creation and distribution	67	33
Item 38	Sharing Images	88	100
Item 39	E-portfolios	100	100
Item 40	Podcasts	100	100

Finally, 26 items (Table 3) were approved as questions for an instrument to assess self-efficacy in effective e-teaching. All the questions were designed on a 4-point Likert scale, ranging from ‘very competent’ to ‘not at all competent’ (rated 4–1).

**Table 3:** Final Items of the Valid and Reliable Questionnaire.

Area of competence	
**1**	I use emerging educational technologies effectively to provide richer learning experiences for students.
**2**	I design and use creative technology-based methods and tools for student assessment.
**3**	I use research-based evidence to make decisions regarding the use of educational technologies in my courses and projects.
**4**	I adhere to relevant standards when designing and using educational technologies (such as professional ethics, intellectual property rights, and related principles).
**5**	I strive to remain up to date regarding the use of emerging educational technologies.
**Open question: Please write any comments or suggestions regarding your ability to identify, design, and effectively use educational technologies for facilitating and assessing student learning:**	
**I can effectively use educational technologies to help students achieve the following outcomes:**	
**6**	Deep thinking
**7**	Participating in discussions about challenging and complex issues
**8**	Reflecting on personal values and how they influence individual actions
**9**	Effective communication with peers, patients, families, nursing colleagues, and other members of the healthcare team
**10**	Acting in the role of a researcher
**11**	Effective information management
**12**	Being creative and innovative
**13**	Identifying personal strengths and areas requiring improvement
**Open question: General comments regarding your ability to use educational technologies to help students achieve program outcomes:**	
**I can use educational technologies effectively to implement the following principles of good teaching:**	
**14**	Providing meaningful interaction between students and instructors
**15**	Encouraging cooperation and reciprocal interaction among students
**16**	Engaging students in active learning
**17**	Providing prompt feedback to students
**18**	Providing adequate time for completing tasks, projects, and assignments.
**19**	Communicating expected outcomes and expectations to students
**Open question: General comments regarding your ability to use educational technologies for effective implementation of “good teaching principles”:**	
**Please evaluate your ability to use the following technologies for creating effective learning experiences:**	
**20**	Using narrated slides with PowerPoint or similar tools
**21**	Using discussion forums and chat rooms in virtual learning environments
**22**	Using graphic organizers or concept mapping tools (such as PowerPoint, Visio, etc.)
**23**	Using virtual storage spaces and file management systems for large project files (such as Google Drive, etc.)
**24**	Using web conferencing tools (such as Zoom, AdobeConnect, etc).
**25**	Creating podcasts
**26**	Using electronic portfolio systems
**General comments regarding your ability to use specific tools to create effective learning experiences in online environments:**	

### Exploratory factor analysis

The data was analyzed with exploratory factor analysis, employing Principal Axis Factoring and Varimax rotation. Before factor analysis, Kaiser-Meyer-Olkin (KMO) and Bartlett tests were used to assess sampling adequacy. The KMO test yielded a value of 0.863, which is considered good to excellent [36,37]. Bartlett's test of sphericity was significant (p < 0.001), indicating that the correlation matrix is not a single matrix and that factor analysis is warranted. Principal axis factoring with Varimax rotation resulted in a five-factor solution. The five extracted factors accounted for 58.046% of the total variance. The first factor accounted for 38.263% of the variance before rotation, and the remaining factors accounted for 8.151%, 4.906%, 3.397%, and 3.329%, respectively. Table 4 shows the initial eigenvalues, the variance explained by each factor before and after extraction, and the variance explained after rotation.

**Table 4:** Factor Extraction and Variance Explained.

Factor	Initial Eigenvalues	% of Variance (Initial)	% of Variance (Extraction)	% of Variance (Rotation)
1	10.354	39.822	38.263	16.504
2	2.564	9.861	8.151	15.072
3	1.621	6.234	4.906	11.164
4	1.272	4.894	3.397	9.281
5	1.258	4.837	3.329	6.025
Total	–	65.648	58.046	69.15

The first factor accounts for the largest proportion of variance (38.26%), while the subsequent factors account for 8.15%, 4.91%, 3.39%, and 3.33%, respectively.

### Factor loadings matrix

After varimax rotation, the factor loadings are in Table 5.

**Table 5:** The rotated factor matrix.

Variable	Factor 1	Factor 2	Factor 3	Factor 4	Factor 5
Item 1				**.539**	
Item 2		0.332		**0.530**	
Item 3	0.496			**0.633**	
Item 4	**0.393**	0.386			
Item 5				**0.737**	
Item 6	**0.596**			0.377	
Item 7	**0.699**	0.425			
Item 8	**0.613**	0.490			
Item 9	**0.521**				
Item 10	**0.659**			0.377	
Item 11	**0.630**				
Item 12	**0.554**	0.401			
Item 13	**0.644**	0.309			
Item 14	0.326	**0.458**			0.303
Item 15	0.330	**0.652**			
Item 16		**0.731**	0.338		
Item 17		**0.748**			
Item 18		**0.654**		0.311	
Item 19	0.359	**0.631**			
Item 20			**0.564**		
Item 21			**0.704**		
Item 22			**0.702**		
Item 23			**0.773**		
Item 24			**0.593**		
Item 25			0.321		**0.554**
Item 26					**0.829**

### Confirmatory factor analysis

Confirmatory Factor Analysis (CFA) was conducted using AMOS (IBM SPSS, Amos 24.0) to evaluate the construct validity of the questionnaire (S1 Fig). The final measurement model comprised five first-order factors. Given the substantial conceptual overlap between the dimensions concerning the “pedagogical use of educational technology” and “technology-supported good teaching practices”, a higher-order factor was introduced to represent their shared underlying construct while retaining the two domains as theoretically distinct first-order factors. The final model demonstrated acceptable fit: χ2(285) = 410.374, p < .001, df = 1.440. The comparative fit index (CFI) was 0.908, while the Tucker–Lewis index (TLI) was 0.895. The root mean square error of approximation (RMSEA) was 0.068, with a 90% confidence interval of 0.053–0.082 and a PCLOSE value of 0.029. The standardized residual-based index, RMR, was 0.030. The GFI and AGFI were 0.776 and 0.724, respectively.

All standardized factor loadings were statistically and substantively acceptable, ranging from 0.559 to 0.856. The standardized loadings for the first-order factors were 0.559–0.747 for Factor 1, 0.654–0.825 for Factor 2, 0.627–0.761 for Factor 3, 0.670–0.816 for Factor 4, and 0.728–0.856 for Factor 5. These findings indicate that all observed items contributed meaningfully to their respective latent constructs, with no standardized loading below 0.50. All factor loadings were statistically significant at p < .001.

The final model included 66 freely estimated parameters and 285 degrees of freedom. The Akaike information criterion (AIC) was 542.374, and the expected cross-validation index (ECVI) was 5.650 (90% CI: 5.130–6.253). These indices were used primarily for comparative purposes in evaluating alternative model specifications, with lower values indicating greater parsimony and better expected comparative performance.

Construct reliability and convergent validity were assessed using composite reliability (CR) and average variance extracted (AVE). Composite reliability values ranged from 0.773 to 0.898, exceeding the commonly recommended threshold of 0.70 for all factors and indicating satisfactory internal construct reliability. AVE values ranged from 0.482 to 0.631. The AVE values for Factors 2, 4, and 5 exceeded the conventional threshold of 0.50. Although the AVE values for Factors 1 and 3 were marginally below 0.50 (0.497 and 0.482, respectively), both constructs demonstrated satisfactory composite reliability and acceptable standardized factor loadings. Taken together, the findings provided generally acceptable evidence of convergent validity, with Factors 1 and 3 demonstrating borderline but defensible convergent validity.

### Questionnaire Reliability

The Cronbach’s alpha coefficient of the current Persian version of the DUSAETCS questions was 0.92, showing excellent internal consistency. All the subscales showed satisfactory internal consistency (Table 6).

**Table 6:** Final reliability.

Cronbach alpha	Assessment Topic
0.81	Area of Competency
0.89	Effectively Use Educational Technologies to Help Students Achieve Outcomes
0.88	Use Educational Technologies to Implement Principles of Good Teaching Effectively
0.79	Create Effective Learning Experiences
0.92	DUSAETCS Tool Total Score

## Discussion

In this study, we aimed to assess the validity and reliability of the Persian version of the DUSAETCS. The findings indicated that the translation was of high quality, as confirmed by experts in specialized language and e-learning. Qualitative face validity was established based on the appearance of the questions, and a panel of experts verified the qualitative content's validity. The CVI and CVR scores demonstrated acceptable content validity. Consequently, the DUSAETCS is a straightforward, valid, and reliable instrument for assessing e-teaching skills.

Various factors influencing self-efficacy in online learning environments include: online learning experience and knowledge, feedback and rewards, online communication and interactions, social influence, and learner motivation and attitude [38]. In this questionnaire, the competency domain comprises items that require an instructor not only to know the tools and technologies but also to use them to achieve learning outcomes [39]. In the domain of using technologies to help students, if an instructor does not believe that using technology can help improve students’ deep thinking skills, reflection, creativity, playing the role of a researcher, and information management, the likelihood of using it purposefully decreases [40]. Items related to effective communication and participation in challenging discussions measure the role of technology in social and professional learning (especially in the field of health and nursing). In the domain of practical teaching principles, studies show that self-efficacy is positively associated with the ability to design and implement active and meaningful learning activities [41]. Items such as meaningful teacher-student interaction, active learning, prompt feedback, and student collaboration indicate the extent to which an individual can implement the principles of effective teaching [42]. In general, this questionnaire covers the factors that influence self-efficacy in the online environment.

The questionnaire items in the context of previous research are as follows

### Area of competency

The competence domain in this questionnaire focuses on the individual's belief in their ability to design, select, use, adhere to ethical standards, and evaluate educational technologies for e-teaching. These items have been emphasized in previous studies, which are discussed below: The TPACK framework, which emphasizes the integration of content knowledge, pedagogy, and technology in a technological environment [43]. According to a systematic review of technology-based assessments across more than 30 countries, these assessments help reduce cheating, increase learning, support supervision, improve teaching, and reduce the workload. They also improve the measurement accuracy, interpretability, interaction, and teacher-parent communication. Therefore, the instructor must be self-efficacious in this area [44]. The findings of Georgiou’s study indicated that self-efficacy beliefs are strong predictors of the extent to which instructors implement evidence-based teaching (EBT) [45]. Observing standards related to professional ethics and intellectual property rights in virtual teaching, given that materials can be immediately copied and shared, is one of the main features of the professional identity of faculty members [46]. Faculty members’ belief in a greater willingness to learn and educational innovation is associated with increased technological self-efficacy [47].

### Helping students achieve

This domain includes items that do not simply measure technical skills in virtual teaching but rather cover deeper dimensions of self-efficacy, including critical thinking, professional reflection, interprofessional communication, research, knowledge management, innovation, and self-assessment. The teacher's belief in their ability to design assignments and activities that stimulate analytical thinking, reasoning, and critical dialogue plays a decisive role in the use of online interactive strategies and persistence in the face of virtual learning [48]. Reflection on personal values and how they influence one’s actions. Recent studies have reported that using different technologies in different ways can reveal the implicit beliefs of faculty and students, enhance teachers’ understanding of cultural diversity, and promote reflection, with implications for teachers’ reflection and belief change [49]. In one study, self-efficacy was the most significant predictor of enhanced social and digital literacy skills [50]. The research-based education approach and evidence indicate that instructor self-efficacy in virtual teaching includes supporting inquiry-based learning [51]. Faculty self-efficacy in e-teaching is significantly associated with effective information management, as well as with positive attitudes toward ICT integration. It is related to the use of technology [52]. Self-efficacy has a positive and direct effect on teacher innovation and creativity [53]. considered the Self-efficacy of faculty members in virtual education related to their belief in making a difference in students’ learning outcomes, which can help students identify strengths and areas for improvement [54].

### Implementing principles of good teaching

Regarding the area “Use Educational Technologies to Implement Principles of Good Teaching Effectively,” Implementing technology to support the seven principles of effective online teaching, including student–teacher communication, student collaboration, active learning, prompt feedback, adequate time on task, high expectations, and respect for diverse talents and learning styles [55].

### Creating learning experiences

The last area, which includes faculty members’ use of educational tools and technologies in teaching, focuses not only on technical mastery of using tools, but also on self-efficacy in selecting, combining, and applying technology tools in accordance with learning objectives, the nature of the content, and the needs of students, which is recognized in previous studies as one of the key components of virtual teaching quality [56].

Another study confirmed the validity of the DUSAETCS in English [28]. It was first utilized by Richter, who received permission on March 23, 2014. The tool is based on Chickering and Gamson’s seven principles for good practice in undergraduate education. It was not initially developed for research purposes; thus, Richter established its validity and reliability for the first time, with a CVI of 0.69. The reliability, as indicated by Cronbach’s coefficient alphas for the scales of the DUSAETCS, was as follows: area of competency (α = 0.915), helping students achieve (α = 0.900), implementing principles of good teaching (α = 0.857), and creating learning experiences (α = 0.903).

Other studies, development, and psychometric exploration of the online Teaching Self-efficacy Inventory by Kevin P. Gosselin developed the OTSEI (Online Teaching Self-Efficacy Inventory) questionnaire that is divided into five main domains of skills: a)Selection of technological resources, b)Virtual interaction, c)Unit content migration, d)Online course alignment, e) Web-based unit structure [7]. The questionnaire was not available free of charge and could not be used without proper authorization. A recent study has provided the “Self-efficacy scale for university teaching in virtual environments, SSUTVE.” The scale offered sufficient indicators to fit a structure composed of nine interrelated factors: basic technology skills, safety in virtual classrooms, ethical-legal aspects, guidance and/or advice in the use of technology resources (related to self-efficacy in digital competencies) and planning, teaching, group management, subject mastery, and evaluation and feedback (related to self-efficacy in teaching competencies). Furthermore, the reliability of the scores and constructs was acceptable [57]. This questionnaire shared similarities in technology use, evaluation, and feedback with the one used in this study.

The current study’s results align with previous studies; for instance, some questions are consistent with the TSES student engagement subscale [34]. But DUSAETCS extends this to technology-based principles, such as rapid feedback through digital tools. Additionally, the theoretical model that best fits virtual education is TPACK-SeS [58], which integrates knowledge types with self-efficacy. It aligns with items in DUSAETCS, such as the development of technology-enriched learning environments. Items like “effective information management” and “being creative and innovative” reflect commonalities between DUSAETCS and TPACK. However, TPACK is science-based, whereas DUSAETCS is practical and lacks explicit measures of content knowledge. In the ethical-legal domain, the SSUTVE overlaps with items from the DUSAETCS questionnaire concerning adherence to high-quality standards (e.g., netiquette, copyright). The computer uses and student engagement domain of the MNESEOT [22] questionnaire includes items that are implicitly linked to specific tools, such as WebEx conferencing. Additionally, the item “How much can you engage students in active learning” from the DUSAETCS domain of good teaching is also relevant.

Generally, our detailed statistical analyses indicated that all questions on the Persian version of the DUSAETCS were valid. The reliability analyses of that version yielded high values, indicating its reliability. Hence, this study established the validity and reliability of the Persian version of DUSAETCS. Finally, a key finding of the current study is that the self-efficacy properties of a novel and beneficial questionnaire have been evaluated among Iranian faculty members.

## Conclusions

Given its excellent reliability, convergent validity, brevity, and ease of implementation, the Persian version of the DUSAETCS can be used to evaluate faculty members’ competence in e-learning and educational technology. As the use of electronic education and educational technologies continues to grow, the results of this study are crucial for supporting faculty members’ self-expression in virtual teaching environments.

### Limitations and directions for future research

This study has two limitations. First, convenience sampling and the relatively small sample of 92 faculty members may have limited the stability and generalizability of the factor solution. Although the KMO value and Bartlett’s test supported the factorability of the data, the sample size was modest for a 26-item instrument and for the estimation of a five-factor CFA model.

Secend the AVE values for two factors were marginally below 0.50, indicating borderline convergent validity for these dimensions. Further research should examine convergent validity, discriminant validity, criterion-related validity, measurement invariance across relevant groups, and responsiveness to changes following faculty development interventions.

## Use of artificial intelligence in article writing

Authors confirm that they use of ChatGPT (OpenAI, July 2026 version, https://chat.openai.com) and Grammarly (https://www.grammarly.com/) for editing and refining the English language in this manuscript. All suggestions were reviewed, and the final text reflects our scholarly voice.

## Supporting information

S1 File Duke University self-assessment educational technology competencies questionnaire. (DOCX)

S2 File Persion version of the questionnaire. (DOCX)

S3 File Study dataset. (XLSX)

S1 Fig Construct validity of the questionnaire. (TIF)
