# Supplementary material for: Psychometric assessment of the Persian version of the DUSAETCS questionnaire: Duke University Self-Assessment Educational Technology Competencies
Source: PLoS One. 2026 Nov 6;21(11):e0360635. doi: 10.1371/journal.pone.0360635 (PMC13742877; doi:10.1371/journal.pone.0360635)
Supplement: Supplementary file 1 [file pone.0360635.s001.docx]

**Duke University Self-Assessment Educational Technology Competencies Questionnaire**

Please rate yourself on each of the following statements that relate to your ability to use educational technologies to facilitate student learning effectively.

*Use the following scale:*

*4 = I am very competent in this area and need little or no guidance*

*3 = I am somewhat competent in this area and will seek out guidance as needed*

*2 = I am not particularly competent in this area and would benefit from guidance from colleagues*

*1 = I am not at all competent in this area and need extensive guidance and support*

| Area of competence | | | | Self-rating | | | | | | |  |
| --- | --- | --- | --- | --- | --- | --- | --- | --- | --- | --- | --- |
| 1 | I utilize new educational technologies to provide my students with richer learning experiences. | | | 1 | | 2 | | | 3 | 4 |  |
| 2 | I design and use innovative technology-based methods and tools to assess my students. | | | 1 | | 2 | | | 3 | 4 |  |
| 3 | We utilize research-based evidence to inform decisions about incorporating educational technology into lessons and projects. | | | 1 | | 2 | | | 3 | 4 |  |
| 4 | In designing and using educational technologies, I adhere to relevant standards (e.g., professional ethics and intellectual property rights, etc). | | | 1 | | 2 | | | 3 | 4 |  |
| 5 | I strive to stay up to date with the latest educational technologies. | | | 1 | | 2 | | | 3 | 4 |  |
| If you are confident in your ability to identify, design, and effectively use educational technology for facilitation and assessment, please provide any questions or comments. | | | | | | | | | | |  |
| I can effectively use educational technologies to help students achieve the following outcomes | | | | | | | | | | | |
| 6 | | Deep thinking | | 1 | | 2 | | | 3 | 4 | |
| 7 | | Participating in discussions about challenging and complex issues | | 1 | | 2 | | | 3 | 4 | |
| 8 | | Reflecting on personal values and how they influence one 's actions | | 1 | | 2 | | | 3 | 4 | |
| 9 | | Communicate effectively with peers, patients, their families, nursing colleagues, and other healthcare team members. | | 1 | | 2 | | | 3 | 4 | |
| 10 | | Acting as a researcher ( Performing the role of researcher) | | 1 | | 2 | | | 3 | 4 | |
| 11 | | Effective information management | | 1 | | 2 | | | 3 | 4 | |
| 12 | | Being creative and innovative | | 1 | | 2 | | | 3 | 4 | |
| 13 | | Identifying strengths and areas for improvement in yourself | | 1 | | 2 | | | 3 | 4 | |
| General comments about your ability to use educational technologies to help students achieve program outcomes | | | | | | | | | | | |
| I can use educational technologies to effectively implement " Principles of Good Teaching " in the following cases | | | | | | | | | | | |
| 14 | | Providing meaningful interaction between students and professors | | 1 | | 2 | | | 3 | 4 | |
| 15 | | Encouraging collaboration and interaction among students | | 1 | | 2 | | | 3 | 4 | |
| 16 | | Engaging students in active learning | | 1 | | 2 | | | 3 | 4 | |
| 17 | | Providing quick feedback to students | | 1 | | 2 | | | 3 | 4 | |
| 18 | | Providing adequate time for completing tasks, projects, and assignments. | | 1 | | 2 | | | 3 | 4 | |
| 19 | | Communicating the desired expectations to the student | | 1 | | 2 | | | 3 | 4 | |
| Write your overall comments about your ability to use educational technologies to implement the " Principles of Good Teaching effectively" : | | | | | | | | | | | |
| Row | | Demonstrate your ability to use the following to provide experienceEvaluate effective learning : | | | | | | | | | |
| 20 | | Using audio-recorded slides with PowerPoint or similar tools | 1 | | 2 | | 3 | 4 | | | |
| 21 | | Using discussion rooms in virtual software | 1 | | 2 | | 3 | 4 | | | |
| 22 | | Using graphic organizing tools or a concept map (such as PowerPoint, Visio, etc.) | 1 | | 2 | | 3 | 4 | | | |
| 23 | | Using virtual storage spaces and file management for large project files ( such as Google Drive, etc. ) | 1 | | 2 | | 3 | 4 | | | |
| 24 | | Using web conferencing tools ( such as Zoom, AdobeConnect, etc). | 1 | | 2 | | 3 | 4 | | | |
| 25 | | Podcast production | 1 | | 2 | | 3 | 4 | | | |
| 26 | | Use of electronic portfolio systems | 1 | | 2 | | 3 | 4 | | | |
| Write your overall opinion about the ability to use specific tools to create a practical learning experience in online environments | | | | | | | | | | | |
