# Supplementary material for: Psychometric assessment of the Persian version of the DUSAETCS questionnaire: Duke University Self-Assessment Educational Technology Competencies
Source: PLoS One. 2026 Nov 6;21(11):e0360635. doi: 10.1371/journal.pone.0360635 (PMC13742877; doi:10.1371/journal.pone.0360635)
Supplement: Supplementary file 2 [file pone.0360635.s002.docx]

بسمه تعالی

خودارزیابی توانمندی فناوری آموزشی

لطفاً به هر یک از عبارات زیر که به توانایی شما در استفاده مؤثر از فناوری‌های آموزشی به منظور تسهیل یادگیری دانشجویان مربوط می‌شود، امتیاز دهید. از مقیاس زیر استفاده کنید:

**4-** **من در این زمینه بسیار توانمند هستم و نیاز به راهنمایی کمی دارم یا اصلا نیازی به راهنمایی ندارم.**

**3-** **من تا حدودی در این زمینه توانمند هستم و در صورت نیاز به دنبال راهنمایی خواهم بود.**

**2-** **من در این زمینه توانمندی خاصی ندارم و از راهنمایی همکاران بهره خواهم برد.**

**1-من اصلاً در این زمینه توانمندی ندارم و نیاز به راهنمایی و حمایت کامل دارم.**

| ردیف | حوزه توانمندی | رتبه بندی خود | | | |
| --- | --- | --- | --- | --- | --- |
|  |  | 1 | 2 | 3 | 4 |
| 1 | از فناوری‌های آموزشی نوین برای فراهم کردن تجارب یادگیری غنی تر برای دانشجویان به بهترین نحو استفاده می‌کنم. |  |  |  |  |
| 2 | برای ارزیابی دانشجویان، روش‌ها و ابزارهای خلاقانه مبتنی بر فناوری طراحی کرده و از آنها استفاده می‌کنم. |  |  |  |  |
| 3 | از شواهد مبتنی بر پژوهش برای تصمیم گیری در مورد استفاده از فناوری آموزشی در درسها و پروژه هایم کمک می گیرم. |  |  |  |  |
| 4 | در طراحی و استفاده از فناوری‌های آموزشی، استانداردهای مربوطه (مانند اخلاق حرفه‌ای، حق مالکیت معنوی و غیره) را رعایت می‌کنم. |  |  |  |  |
| 5 | تلاش می‌کنم در زمینه استفاده از فناوری‌های نوین آموزشی به روز باشم. |  |  |  |  |
| چنانچه در مورد توانایی خود در شناسایی، طراحی و استفاده موثر از فناوری آموزشی برای تسهیل و ارزیابی یادگیری دانشجویان نکته یا مطلبی دارید، بنویسید: | | | | | |

| ردیف | من می‌توانم به طور مؤثر از فناوری‌های آموزشی برای کمک به دانشجویان برای دستیابی به پیامدهای زیر استفاده کنم: | رتبه بندی خود | | | |
| --- | --- | --- | --- | --- | --- |
|  |  | 1 | 2 | 3 | 4 |
| 6 | تفکر عمیق |  |  |  |  |
| 7 | مشارکت در بحث درباره مسایل پرچالش و دشوار |  |  |  |  |
| 8 | تامل بر ارزش‌های شخصی و نحوه تأثیرگذاری آنها بر اعمال فرد |  |  |  |  |
| 9 | ارتباط موثر با همتایان، بیماران، خانواده‌ها، همکاران پرستار و سایر اعضای تیم مراقبت سلامت |  |  |  |  |
| 10 | عمل کردن در نقش محقق (ایفای نقش محقق) |  |  |  |  |
| 11 | مدیریت مؤثر اطلاعات |  |  |  |  |
| 12 | خلاق و نوآور بودن |  |  |  |  |
| 13 | شناسایی نقاط قوت و زمینه‌های نیاز به بهبود در خود |  |  |  |  |
| نظرات کلی در مورد توانایی شما در استفاده از فناوری‌های آموزشی برای کمک به دانشجویان در دستیابی به نتایج برنامه: | | | | | |

| ردیف | من می‌توانم از فناوری‌های آموزشی برای اجرای موثر "اصول تدریس خوب" در موارد زیر استفاده کنم: | رتبه بندی خود | | | |
| --- | --- | --- | --- | --- | --- |
|  |  | 1 | 2 | 3 | 4 |
| 14 | فراهم کردن مواجهه معنی دار بین دانشجویان و استاد |  |  |  |  |
| 15 | تشویق به همکاری و تعامل متقابل بین دانشجویان |  |  |  |  |
| 16 | درگیر کردن دانشجویان در یادگیری فعال |  |  |  |  |
| 17 | ارائه بازخورد سریع به دانشجویان |  |  |  |  |
| 18 | فراهم کردن مدت زمان مناسب برای انجام وظایف، پروژه‌ها و تکالیف درسی. |  |  |  |  |
| 19 | انتقال انتظارات مورد نظر به دانشجویان |  |  |  |  |
| نظرات کلی خود را در باره توانایی شما در استفاده از فناوری‌های آموزشی برای اجرای موثر "اصول تدریس خوب" بنویسید: | | | | | |

| ردیف | توانایی خود را برای استفاده از موارد زیر به منظور فراهم سازی تجربیات یادگیری موثر ارزیابی کنید: | رتبه بندی خود | | | |
| --- | --- | --- | --- | --- | --- |
|  |  | 1 | 2 | 3 | 4 |
| 20 | استفاده از اسلاید صدا گذاری شده با پاورپوینت یا ابزارهای مشابه |  |  |  |  |
| 21 | استفاده از اتاقهای بحث و گفتگو در نرم افزارهای مجازی |  |  |  |  |
| 22 | استفاده از ابزارهای سازماندهنده گرافیکی یا نقشه مفهومی(مانند پاورپوینت، ویزیو و...... |  |  |  |  |
| 23 | استفاده از فضاهای ذخیره مجازی و مدیریت فایل برای فایلهای پروژه‌های بزرگ(مانند گوگل درایو و ....) |  |  |  |  |
| 24 | استفاده از ابزارهای وب کنفرانس (مانند زوم، ادوبی کانکت و ...) |  |  |  |  |
| 25 | تهیه پادکست |  |  |  |  |
| 26 | استفاده از سامانه های پورتفولیوی الکترونیکی |  |  |  |  |
| نظر کلی خود را در مورد توانایی استفاده از ابزارهای خاص برای ایجاد تجربه یادگیری موثر در محیط‌های انلاین بنویسید: | | | | | |
